# Supplementary material for: Higher surface folding of the human premotor cortex is associated with better long-term learning capability
Source: Commun Biol. 2024 May 25;7:635. doi: 10.1038/s42003-024-06309-z (PMC11127997; doi:10.1038/s42003-024-06309-z)
Supplement: Supplementary file 2 — Supplemental Material [file 42003_2024_6309_MOESM2_ESM.pdf]

Supplementary Text

Regional specificity of the association between cortical folding and learning rate

Cortical folding develops mostly prenatally and during the first years of life [1]. Therefore, cortical developmental events preceding the late maturation phase could have an impact on cortical folding in pre-SMA/SMA and its relation to learning ability. To rule out factors that influenced the extent of cortical folding of the whole hemisphere, we re-examined the correlation between local cortical folding in pre-SMA/SMA and the learning rate (R^2 = 0.317, p < .001, Fig. 2B), this time adjusted for individual differences in the folding of the whole cortex (total folding index, see [2]). Although a higher total folding index was significantly correlated with a steeper learning rate (R^2 = 0.05, p = .041), the relationship between local cortical folding in pre-SMA/SMA and learning ability was only slightly reduced when we adjusted for total folding index (partial R^2 = 0.286, p < .001). This shows that the impact of cortical folding on learning is region-specific and relatively independent from hemispheric cortical folding.

Effect size estimation for interventions using correction for individual differences in folding

Quantitative comparison of effect sizes suggests that performance-enhancing psychological strategies (positive social comparative feedback [R^2 = 0.31 in [3]]) or exercise interventions (post-exercise physical activity [R^2 = 0.301 in [4] or chronic physical activity [R^2 = 0.259 in [5]]) can have as large an impact on stabilometer motor performance as cortical folding in the pre-SMA/SMA (R^2 = 0.30). Our previous study [5] showed that 2 weeks of intense physical exercise prior to motor practice increases the learning rate in this postural task. Since we included this subsample in our analysis (e.g., the green dots in Fig. 2C), we were interested in the covariation pattern of cortical folding and previous physical exercise effects on learning rate. Therefore, we reanalyzed the data and, first, replicated the positive effect of prior intense physical activity on learning rate compared to the control condition (two-tailed unpaired t-test, $t(29)$ = 2.50, p = .022, mean difference = 0.12, Cohen's d = 0.898, 95% CI = 0.150, 1.632). When we adjusted for inter-individual differences in cortical folding in pre-SMA/SMA, the magnitude of the exercise effect continued to increase (two-tailed unpaired t-test, $t(29)$ = 3.29, p = .003, mean difference = 0.13, Cohen's d = 1.181, 95% CI = 0.406, 1.940), indicating independent effects of prior intense exercise and cortical folding on learning rate. From a statistical perspective, this finding suggests that consideration of individual predispositions in the brain can improve effect size estimation for interventions targeting behavioral change.

The impact of motivation

Feedback and instructions from teachers or coaches can have a positive (or negative) impact on motor performance and learning [6]. Modulating a learners' focus-of-attention or motivation can be beneficial for skill acquisition and retention [7]. Although we controlled for performance feedback as well as task instructions and the degree of practice exposure throughout our experiments, individual differences in motivation may have had an impact on motor performance. However, core motivational brain circuits are localized in the midbrain, ventral striatum and orbitofrontal cortex [8] and neural activity in these areas adapts during learning [9]. Future studies are required to disentangle the motivational contribution of reward-related brain structures to motor learning of the stabilometer task. Of particular interest in this context is whether individual differences in motivation share variance with the effects of cortical folding on learning rate. The spatial localization of our present neuroimaging result, however, indicates that cortical folding in pre-SMA/SMA supports postural task-specific learning capabilities rather than general functions such as motivation.

Relationship between cortical curvature and training-induced microstructural cortical plasticity

The sample used for the main analysis consists of three subsamples (see Supplementary Table 4) with slightly different research questions and study designs. Respective research questions centered around balance-induced and exercise-induced brain plasticity and the time course of training-related functional and structural brain changes. In some of these studies, and thus also in the sample for our main analysis, training-related changes in the left pre-SMA/SMA were identified. For example, in our original balance training study[10,11], we found changes in gray matter volume and functional connectivity in the left pre-SMA/SMA. The pre-SMA/SMA was also observed in our most recent training study[12] (n=26). Both data sets were part of the current sample for the main analysis (n=84). A thorough comparison between cross-sectional measurements of cortical anatomy (e.g., cortical folding) and training-induced changes in cortical structure requires homogenization of different training protocols and different imaging methodologies between studies (partly on different MRI scanners). This is crucial in order to identify common patterns of plasticity across studies, which can then be compared with the baseline values of e.g. cortical folding. In the meantime, however, we can correlate effects within a subsample. Our most recent study[12] provides the largest subsample (n=26) and a plasticity index (orientation dispersion index, ODI) that changed with balance training and also correlated with inter-individual differences in learning rate. While both measures individually correlated with learning rate in this sub-sample ($r = 0.59$ for ODI change and $r = 0.51$ for cortical curvature), a correlation analysis suggests a low correlation between ODI change and cortical folding in pre- SMA/SMA ($r = .18$). When we entered these two variables (change in ODI as well as cortical curvature) in a linear regression model predicting learning rate, the results show that both ODI change and cortical curvature represent two distinct mechanisms significantly predicting individual differences in learning rate ($\beta=.53$, $T=3.3$, $p=.004$ for ODI change; $\beta=.36$, $T=2.2$, $p=.036$ for curvature).

Supplementary Table 1: List of differing head coils, echo times, repetition times, flip angles, and inversion times of the T1-weighted MRI datasets used for the main analysis of learning rate (n=84, samples marked with *) and the additional analysis of the first practice session (n =131).

number of subjects	head coil	echo time (ms)	repetition time (ms)	flip angle (°)	inversion time (ms)
N=27*	32 ch	3,46	1300	10	650
N=26*	64 ch	2,82	2500	7	1100
N=31*	32 ch	2,98	2300	9	900
N=24	32 ch	2,96	2300	9	900
N=11	12 ch	3.46	1300	10	650
N=6	12 ch	2.96	2300	9	900
N=5	32 ch	3,46	1300	10	650
N=1	12 ch	2,23	1300	10	650

Supplementary Table 2: Cortical folding results from the main analysis (N=84) are not dependent on the use of different imaging protocols. Table shows multiple regression models containing cortical folding in pre-SMA/SMA, age, gender, body height, and TIV as predictors of learning rate (Model A). In model B, head coil (binary coded) was included as additional co-regressor, whereas models C considered the three imaging protocols as dummy coded variable (based on Suppl. Table 1, marked with*). Partial regression statistics between cortical folding and learning rate are shown.

	β	T	p
Model A	,398	4,988	<,001
Model B (+ head coil)	,402	4,988	<,001
Model C (+ MRI protocol)	,404	5,335	<,001

Supplementary Table 3: Correlations between cortical folding and learning rate are consistent between the 32-ch (N=58) and 64-ch (N=26) data subsets of the main analysis (see supplementary table 1). We extracted cortical folding values in pre-SMA/SMA for these two subsets of participants. The residuals of learning rate and cortical folding (see Fig. 2 in the main manuscript) constituted the input for Chow's test (Chow, 1960), which tests the null hypothesis that two regression lines are equal (i.e. can be represented by one single regression line).

	F-statistics	p-value
Left pre-SMA/SMA	0.071442	0.9311

Supplementary Table 4: Detailed characteristics of samples included in the main analysis (N=84) and the separate analyses of initial performance and short-term adaptations (N=131). The table includes sub-study, sub-sample, age (mean), number of female participants, body height (in cm) and mean TIV (in ml). Data was analyzed from several studies: [a] from Taubert et al. (2010)[11], [b] unpublished data, [c] from Lehmann et al. (2023)[12], [d] from Lehmann et al. (2020)[5], e from Taubert et al. (2016)[13].

Whole sample	Analysis	Sub-studies	Sub-groups	Mean age	#females	Mean body height (cm)	Mean TIV
N=131	Main sample for analysis of long-term learning (6 practice sessions, N=84)	N=27	N=14[a]	25.8	6	172	1481
			N=13[b]	22.8	4	177	1569
		N=26	N=26[c]	22.1	4	179	1557
		N=31	N=16[d]	23.6	11	174	1505
			N=15[d]	24.3	9	173	1478
	Additional sample with short-term learning data only (1 practice session, N=47)	N=24	N=24[e]	25.9	12	174	1443
		N=11	N=11[b]	26.3	6	173	1502
		N=6	N=6[b]	26.5	2	173	1530
		N=5	N=5[b]	27.4	2	173	1476
		N=1	N=1[b]	23.0	1	163	1338

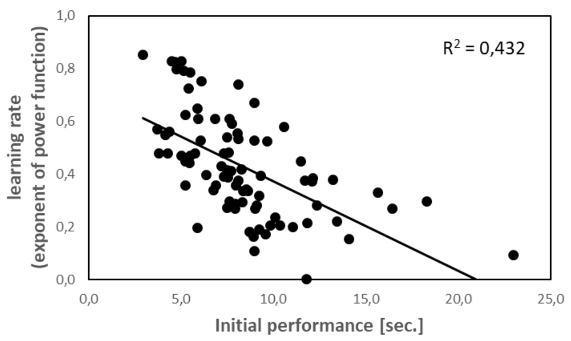

Supplementary Figure 1: Relationship between learning rate and initial level of performance.

Scatter plot showing Spearman correlation between learning rate *n* across the six practice sessions and initial performance in session 1.

		learning rate n	initial performance a	age	gender	height	TIV	study1	study2	study3
learning rate n	correlation coefficient	--								
	N	84								
initial performance a	correlation coefficient	-,657	--							
	Sig. (2-sided)	,000								
	N	84	84							
age	correlation coefficient	-,170	-,037	--						
	Sig. (2-sided)	,881	,741							
	N	84	84	84						
gender	correlation coefficient	,355	-,207	,043	--					
	Sig. (2-sided)	,001	,059	,696						
	N	84	84	84	84					
height	correlation coefficient	,220*	-,210*	-,118	,571	--				
	Sig. (2-sided)	,045	,055	,284	,000					
	N	84	84	84	84	84				
TIV	correlation coefficient	,230*	-,144	,075	,633	,624	--			
	Sig. (2-sided)	,035	,191	,497	,000	,000				
	N	84	84	84	84	84	84			
study1	correlation coefficient	,079	-,045	,276		,274	,155	--		
	Sig. (2-sided)	,441	,687	,011		,012	,159			
	N	84	84	84		84	84	84		
study2	correlation coefficient	-,194*	,070	,171		-,107	,005		--	
	Sig. (2-sided)	,078	,526	,121		,331	,966			
	N	84	84	84		84	84		84	
study3	correlation coefficient	,263	-,025	,099*		-,159*	-,153			--
	Sig. (2-sided)	,016	,881	,370		,149	,164			
	N	84	84	84		84	84			84

Supplementary Figure 2: Correlation matrix including initial performance and learning rate as well as nuisance variables.

Correlation matrix with learning rate *n* and all nuisance variables used in the main analysis (n=84). Coefficients indicate Pearson or Spearman correlations between two continuous variables (e.g. age and body height) depending on data normal distribution as well as Point-biserial correlation coefficients for a continuous and a categorial variable (e.g. gender and age). Correlations between two categorical variables (e.g. gender and study 1) were omitted. Studies 1, 2 and 3 include datasets for the main analysis (N=84) indicated in supplementary table 1 with *.

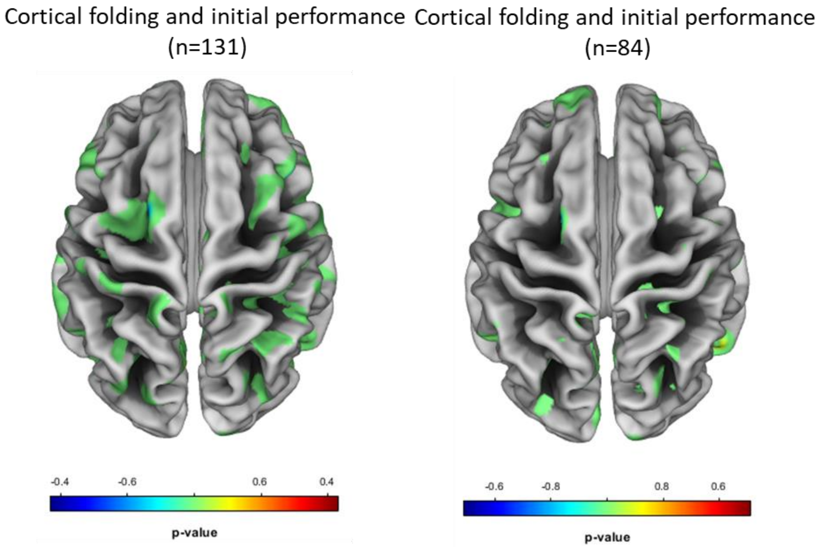

Supplementary Figure 3: Cortical folding and initial performance.

Correlation between local cortical folding and initial performance (mean performance across 15 trials in practice session 1) in 131 (left) and 84 (right) participants (see supplementary table 1). No significant effects were found across the whole cortex. Color bar represents uncorrected p values.

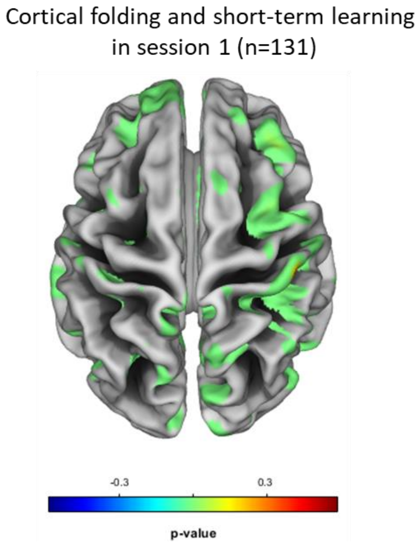

Supplementary Figure 4: Cortical folding and early performance improvements. Correlation between local cortical folding and early (short-term) learning within the first practice session (N=131). No significant effects were found across the whole cortex. Color bar represents uncorrected p values.

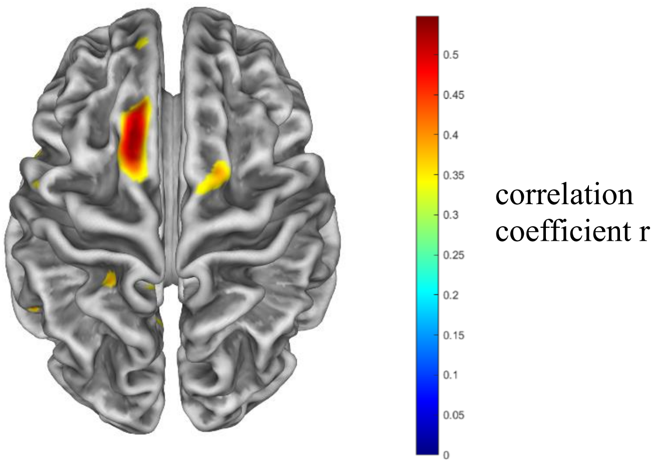

Supplementary Figure 5: Correlation coefficients r depicting positive relationships between cortical folding and learning rate.

Vertex-wise map of correlation coefficients (*r*) from the main exploratory analysis (Fig. 2). The color bar represents *r* values depicting the positive correlation between local cortical folding and learning rate *n*.

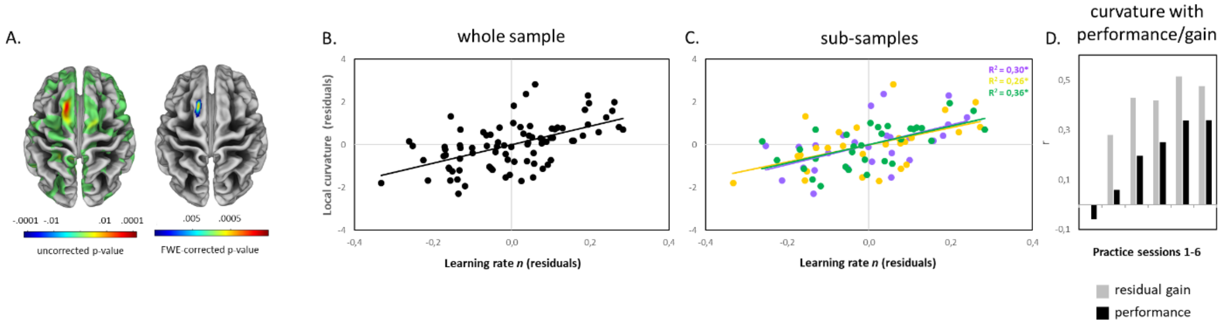

Supplementary Figure 6: Reproducible effect of cortical folding on learning rate.
(A) Results of whole-brain regression of vertex-wise cortical curvature to learning rate using a second MRI scan of the same participants. Uncorrected results at $p < .001$ (left) and family-wise error-corrected results at $p < .05$ (right) were projected onto a template brain showing variations in sulcus depth. (B) Positive correlation of residual cortical curvature (in the cluster representing the FWE-corrected effect in the exploratory analysis [Fig. 2A]) and learning rate. (C) Subsample results in the three independent learning experiments. (D) Pearson correlation coefficients between residualized cortical curvature and motor performance. Grey bars represent session-specific performance controlled for initial performance in session 1 (i.e., residual gain) and black bars represent correlations with actual session-specific performance. * indicate significant correlations at $p < .05$.

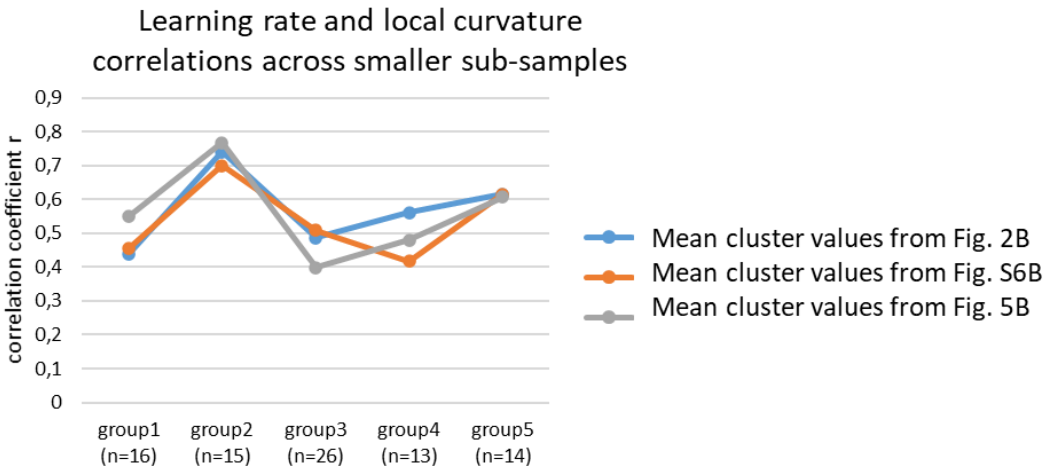

Supplementary Figure 7: Study-specific sub-group analyses of cortical curvature effects on learning rate.

The five sub-groups were drawn from the three longitudinal training studies[5,11,12]. Sub-group analyses of the effect of cortical curvature on learning rate *n*. The effect was consistent across regions-of-interests (ROI) obtained from the original analysis (Fig. 2), its reproduction with another MRI scan of the same participants as well as from a previous study in which postural learning-induced grey matter changes were found in this region [11]. All correlation coefficients were significant at $p < .05$.

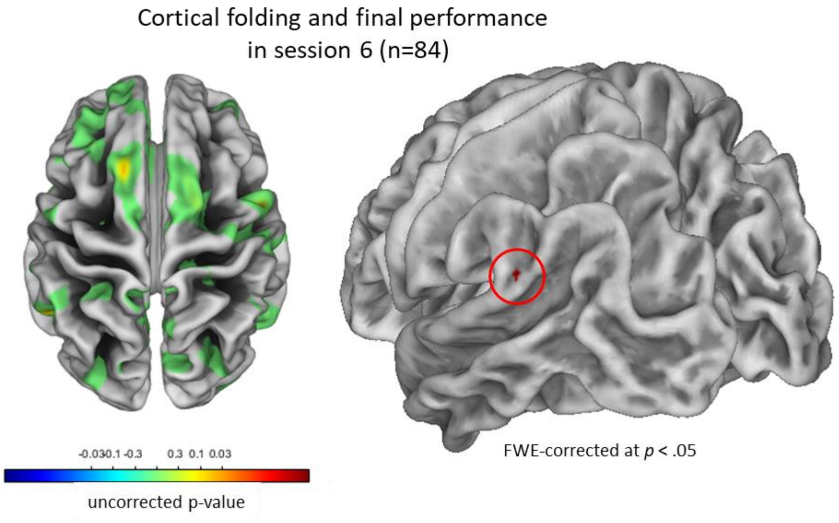

Supplementary Figure 8: Statistical maps showing positive correlations between cortical folding and asymptotic (final) performance.

Correlation between local cortical folding and final performance in the sixth practice session (N=84). Significant effects (corrected for multiple comparisons) were found in a cluster located in the left supramarginal gyrus (see red circle on the right; labeling according to the Desikan-Killiany atlas). Note a trend level effect (left) in the cluster in left pre-SMA/SMA overlapping with the cortical folding effect on learning rate (Fig. 2). Color bar represents uncorrected p values.

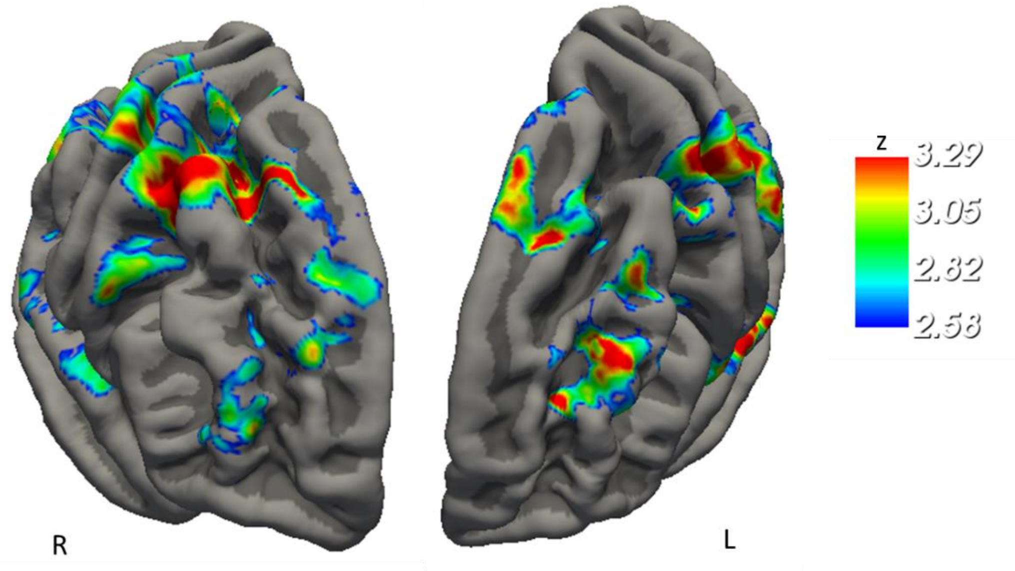

Supplementary Figure 9: Positive correlation between local gyrification index (Schaer et al., 2008) and learning rate.

Vertex-wise correlation between local gyrification index (lGI) and learning rate (N=84). While local curvature characterizes local geometrical properties of the cortical surface (Fig. 2), the lGI is sensitive to variations in surface area buried within cortical sulci. Note the positive correlation in the caudal part of the left superior frontal gyrus spatially coincides with results from the local curvature analysis (pre-SMA/SMA, Fig. 2). Color bar represents Z values.

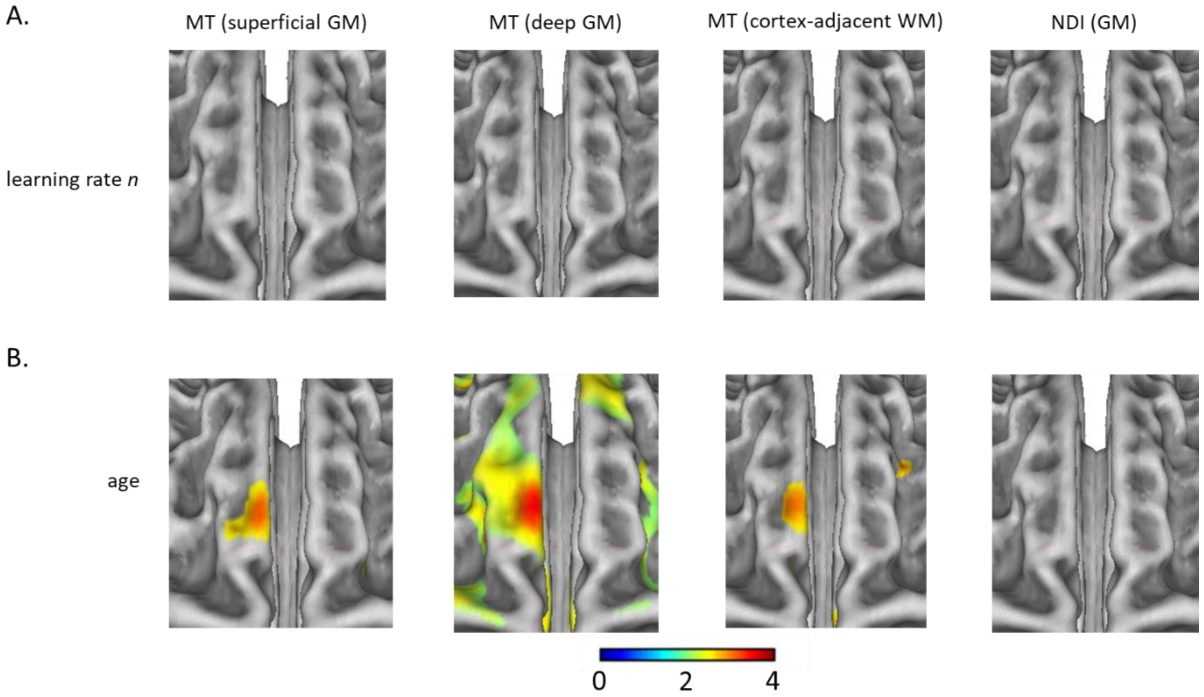

Supplementary Figure 10: Effects of age and learning rate on intracortical microstructure. Correlation analysis of study 2 (N = 26, yellow dots in Fig.2) for which we acquired additional quantitative MRI protocols. We analysed intracortical microstructural properties include myelin-sensitive magnetization transfer (MT). Vertex-wise correlations with MT were calculated with learning rate *n* (A) and age (B). Exploratory statistical thresholds ($p < .01$ uncorrected at vertex-level) were applied and thresholded *t* values are displayed. Color bar represents *t* values.

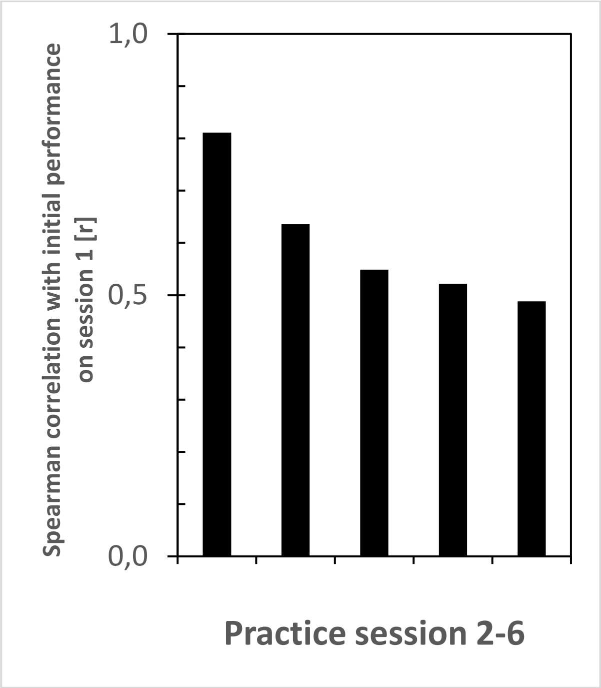

Supplementary Figure 11: The influence of initial performance differences on later achievements decreased with practice.

Pearson correlation between mean performance in session 1 and mean performance in sessions 2-6.

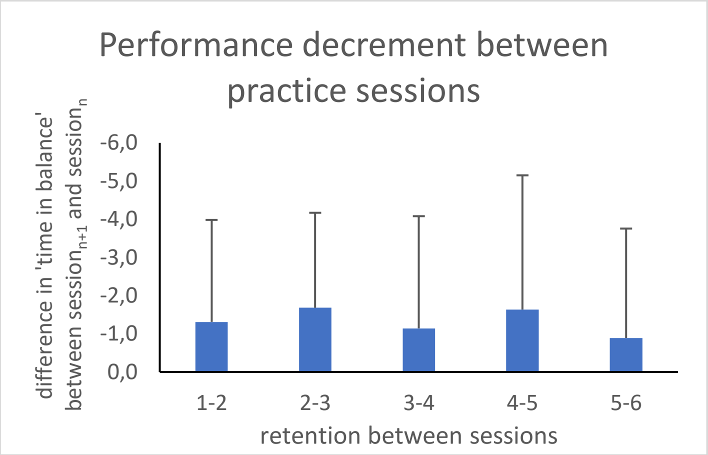

Supplementary Figure 12: Performance decrements between successive practice sessions. The bars represent the difference in our primary behavioural outcome measure (time in target zone in seconds) between the mean of the first two trials of the actual session (session n+1) and the mean of the last two trials of the previous session (session n).

Supplementary References

1. Llinares-Benadero, C. & Borrell, V. Deconstructing cortical folding: genetic, cellular and mechanical determinants. *Nat Rev Neurosci* **20,** 161–176; 10.1038/s41583-018-0112-2 (2019).
2. van Essen, D. C. & Drury, H. A. Structural and Functional Analyses of Human Cerebral Cortex Using a Surface-Based Atlas. *J. Neurosci.* **17,** 7079–7102; 10.1523/JNEUROSCI.17-18-07079.1997 (1997).
3. Lewthwaite, R. & Wulf, G. Social-comparative feedback affects motor skill learning. *Quarterly Journal of Experimental Psychology* **63,** 738–749; 10.1080/17470210903111839 (2010).
4. Wanner, P. *et al.* Acute exercise following skill practice promotes motor memory consolidation in Parkinson's disease. *Neurobiology of Learning and Memory* **178,** 107366; 10.1016/j.nlm.2020.107366 (2021).
5. Lehmann, N., Villringer, A. & Taubert, M. Colocalized White Matter Plasticity and Increased Cerebral Blood Flow Mediate the Beneficial Effect of Cardiovascular Exercise on Long-Term Motor Learning. *J. Neurosci.* **40,** 2416–2429; 10.1523/JNEUROSCI.2310-19.2020 (2020).
6. Schmidt, R. A. & Lee, T. D. *Motor control and learning. A behavioral emphasis.* 5th ed. (Human Kinetics, Champaign, IL, 2011).
7. Lewthwaite, R. & Wulf, G. Optimizing motivation and attention for motor performance and learning. *Current Opinion in Psychology* **16,** 38–42; 10.1016/j.copsyc.2017.04.005 (2017).
8. Schultz, W. Reward Processing in Primate Orbitofrontal Cortex and Basal Ganglia. *Cerebral Cortex* **10,** 272–283; 10.1093/cercor/10.3.272 (2000).
9. Diederen, K. M., Spencer, T., Vestergaard, M. D., Fletcher, P. C. & Schultz, W. Adaptive Prediction Error Coding in the Human Midbrain and Striatum Facilitates Behavioral Adaptation and Learning Efficiency. *Neuron* **90,** 1127–1138; 10.1016/j.neuron.2016.04.019 (2016).
10. Taubert, M., Lohmann, G., Margulies, D. S., Villringer, A. & Ragert, P. Long-term effects of motor training on resting-state networks and underlying brain structure. *NeuroImage* **57,** 1492–1498; 10.1016/j.neuroimage.2011.05.078 (2011).
11. Taubert, M. *et al.* Dynamic Properties of Human Brain Structure: Learning-Related Changes in Cortical Areas and Associated Fiber Connections. *J. Neurosci.* **30,** 11670–11677; 10.1523/JNEUROSCI.2567-10.2010 (2010).
12. Lehmann, N. *et al.* Changes in Cortical Microstructure of the Human Brain Resulting from Long-Term Motor Learning. *J. Neurosci.* **43,** 8637–8648; 10.1523/JNEUROSCI.0537-23.2023 (2023).
13. Taubert, M., Mehnert, J., Pleger, B. & Villringer, A. Rapid and specific gray matter changes in M1 induced by balance training. *NeuroImage* **133,** 399–407; 10.1016/j.neuroimage.2016.03.017 (2016).
