# Supplementary material for: Higher surface folding of the human premotor cortex is associated with better long-term learning capability
Source: Commun Biol. 2024 May 25;7:635. doi: 10.1038/s42003-024-06309-z (PMC11127997; doi:10.1038/s42003-024-06309-z)
Supplement: Supplementary file 3 — Reporting Summary [file 42003_2024_6309_MOESM3_ESM.pdf]

nature portfolio

Corresponding author(s): Marco Taubert

Last updated by author(s): Apr 24, 2024

Reporting Summary

Nature Portfolio wishes to improve the reproducibility of the work that we publish. This form provides structure for consistency and transparency in reporting. For further information on Nature Portfolio policies, see our Editorial Policies and the Editorial Policy Checklist.

Statistics

For all statistical analyses, confirm that the following items are present in the figure legend, table legend, main text, or Methods section.

n/a	Confirmed	
☐	☒	The exact sample size (*n*) for each experimental group/condition, given as a discrete number and unit of measurement
☐	☒	A statement on whether measurements were taken from distinct samples or whether the same sample was measured repeatedly
☐	☒	The statistical test(s) used AND whether they are one- or two-sided<br>*Only common tests should be described solely by name; describe more complex techniques in the Methods section.*
☐	☒	A description of all covariates tested
☐	☒	A description of any assumptions or corrections, such as tests of normality and adjustment for multiple comparisons
☐	☒	A full description of the statistical parameters including central tendency (e.g. means) or other basic estimates (e.g. regression coefficient) AND variation (e.g. standard deviation) or associated estimates of uncertainty (e.g. confidence intervals)
☐	☒	For null hypothesis testing, the test statistic (e.g. *F*, *t*, *r*) with confidence intervals, effect sizes, degrees of freedom and *P* value noted<br>*Give P values as exact values whenever suitable.*
☒	☐	For Bayesian analysis, information on the choice of priors and Markov chain Monte Carlo settings
☒	☐	For hierarchical and complex designs, identification of the appropriate level for tests and full reporting of outcomes
☐	☒	Estimates of effect sizes (e.g. Cohen's *d*, Pearson's *r*), indicating how they were calculated

Our web collection on statistics for biologists contains articles on many of the points above.

Software and code

Policy information about availability of computer code

Data collection	Microsoft Office - Excel 97-2003
Data analysis	Statistical Parametric Mapping (SPM) 12 v7771<br>CAT12 v12.7 r1738<br>spm_TFCE v210<br>Matlab R2020b<br>FreeSurfer 6<br>Microsoft Office - Excel 2010<br>Microsoft Office - PowerPoint 2010<br>R i386 4.1.1<br>RStudio 1.3.1093<br>IBM SPSS Statistics Version 28.0.1.0

For manuscripts utilizing custom algorithms or software that are central to the research but not yet described in published literature, software must be made available to editors and reviewers. We strongly encourage code deposition in a community repository (e.g. GitHub). See the Nature Portfolio guidelines for submitting code & software for further information.

Data

Policy information about availability of data

All manuscripts must include a data availability statement. This statement should provide the following information, where applicable:

- Accession codes, unique identifiers, or web links for publicly available datasets
- A description of any restrictions on data availability
- For clinical datasets or third party data, please ensure that the statement adheres to our policy

All data are available in the main text or the supplementary materials. In addition, data and code used for this project have been made freely available under https://doi.org/10.24352/UB.OVGU-2023-095. Visualizations of all sulcal definitions generated for each participant are provided in the Supplementary Materials. Requests for further information or raw data should be directed to the corresponding author, M.T. (marco.taubert@ovgu.de).

Research involving human participants, their data, or biological material

Policy information about studies with human participants or human data. See also policy information about sex, gender (identity/presentation), and sexual orientation and race, ethnicity and racism.

Reporting on sex and gender	In this study, we include a sample of 131 right-handed participants of both sexes with normal or corrected-to-normal vision (mean age of 24.6 years, age range of 19-35 years, 57 females, mean body height 174 cm, body height range 153-191 cm).
Reporting on race, ethnicity, or other socially relevant groupings	Data on race, ethnicity, or other socially relevant groupings were not obtained in this study.
Population characteristics	In this study, we include a sample of 131 right-handed participants of both sexes with normal or corrected-to-normal vision (mean age of 24.6 years, age range of 19-35 years, 57 females, mean body height 174 cm, body height range 153-191 cm).
Recruitment	Participants were recruited via newspaper advertisements, telephone calls, from university courses and from a larger database of MRI-compatible participants at MPI Leipzig.
Ethics oversight	Ethics Committees of the Universities of Leipzig and Magdeburg (Germany)

Note that full information on the approval of the study protocol must also be provided in the manuscript.

Field-specific reporting

Please select the one below that is the best fit for your research. If you are not sure, read the appropriate sections before making your selection.

☐ Life sciences ☒ Behavioural & social sciences ☐ Ecological, evolutionary & environmental sciences

For a reference copy of the document with all sections, see nature.com/documents/nr-reporting-summary-flat.pdf

Behavioural & social sciences study design

All studies must disclose on these points even when the disclosure is negative.

Study description	quantitative longitudinal intervention study
Research sample	A sample of 131 right-handed participants with normal or corrected-to-normal vision (mean age of 24.6 years, age range of 19-35 years, 57 females, mean body height 174 cm, body height range 153-191 cm) was included from the datasets of three independent motor learning experiments. The sample consisted of young healthy participants that were able to perform and learn the dynamic balance task. Inclusion was constrained to participants eligible to testing in an MRI scanner.
Sampling strategy	Random sampling via newspaper and from an institute-specific database. The sample consisted of multiple sub-samples mostly from previously published studies (but also unpublished sub-samples, please see Supplementary Table 4).
Data collection	Behavioral data was collected with a stabilometer device (Lafayette Instruments) and performance values were recorded via pen and paper or via MS Excel. Different experiementers performed training of participants and only one experimenter was within the training room together with the participant. Experimenters were not blinded to experiemental conditions. MRI data was acquired from technical assistants not involved in the specific study.
Timing	Data from multiple sub-samples were acquired between 2009 and 2018.
Data exclusions	No data from the four original studies were excluded.
Non-participation	We had 3 dropouts due to illness or injury.

Randomization	Participants were either allocated randomly to experimental and control group (Lehmann et al., 2020) or participants were not randomly allocated into groups.

Reporting for specific materials, systems and methods

We require information from authors about some types of materials, experimental systems and methods used in many studies. Here, indicate whether each material, system or method listed is relevant to your study. If you are not sure if a list item applies to your research, read the appropriate section before selecting a response.

Materials & experimental systems

n/a	Involved in the study
☒	☐ Antibodies
☒	☐ Eukaryotic cell lines
☒	☐ Palaeontology and archaeology
☒	☐ Animals and other organisms
☒	☐ Clinical data
☒	☐ Dual use research of concern
☒	☐ Plants

Methods

n/a	Involved in the study
☒	☐ ChIP-seq
☒	☐ Flow cytometry
☐	☒ MRI-based neuroimaging

Plants

Seed stocks	*Report on the source of all seed stocks or other plant material used. If applicable, state the seed stock centre and catalogue number. If plant specimens were collected from the field, describe the collection location, date and sampling procedures.*
Novel plant genotypes	*Describe the methods by which all novel plant genotypes were produced. This includes those generated by transgenic approaches, gene editing, chemical/radiation-based mutagenesis and hybridization. For transgenic lines, describe the transformation method, the number of independent lines analyzed and the generation upon which experiments were performed. For gene-edited lines, describe the editor used, the endogenous sequence targeted for editing, the targeting guide RNA sequence (if applicable) and how the editor was applied.*
Authentication	*Describe any authentication procedures for each seed stock used or novel genotype generated. Describe any experiments used to assess the effect of a mutation and, where applicable, how potential secondary effects (e.g. second site T-DNA insertions, mosiacism, off-target gene editing) were examined.*

Magnetic resonance imaging

Experimental design

Design type	T1-weighted MPRAGE sequence
Design specifications	T1-weighted MPRAGE data were acquired before, during and after the 6-week dynamic balance training or 4 weeks before the onset of training, immediately before training and after 4 week of training.
Behavioral performance measures	Time in balance (in seconds) was the behavioral outcome parameter on each trial. Data from 90 trails (6 sessions) were used to fit a learning curve (power function) with slope and intercept as behavioral variables.

Acquisition

Imaging type(s)	structural
Field strength	3 Tesla
Sequence & imaging parameters	Standard MPRAGE sequence
Area of acquisition	Whole-brain acquisition

Diffusion MRI ☐ Used ☒ Not used

Preprocessing

Preprocessing software	T1-weighted MRI data was preprocessed using CAT12 (v12.7 r1738) running in MATLAB (Matlab R2020b). We used standard preprocessing parameters according to the recommendations of Christian Gaser and colleagues from Uni Jena (https://neuro-jena.github.io/cat/index.html#DOWNLOAD). We applied a surface-based heat kernel filter with FWHM = 20 mm. MR images of all participants passed both the visual quality inspection and the CAT12 data quality checks. All scans from 131 participants reached a weighted average image quality rating (IQR) of 86.79% (range 80.64%–89.87%) corresponding to a

quality grade B while the long-term practice cohort (N=84) reached a weighted average (IQR) of 87.32% (quality grade B; range 85.62%-89.87%). In addition, surface parameters were obtained using FreeSurfer 6 (recon -all command) pipeline in order to analyze cortical surface area, cortical thickness and cortical folding index within gyral and sulcal regions. We supplemented the analysis of local cortical geometry (curvature) with an analysis of a gyrification metric that depends on the ratio between the outer hull surface area and the local cortical surface area (called outer-surface-based gyrification indices). Therefore, we computed the local gyrification index (Schaer et al.) of freesurfer cortical reconstructions.

Normalization

Spatial registration of cortical surface maps was performed in accordance with recommendations from https://neuro-jena.github.io/cat/index.html#DOWNLOAD.

Normalization template

FsAverage template

Noise and artifact removal

Image quality control was performed using CAT12 data quality tools. All scans from 131 participants reached a weighted average image quality rating (IQR) of 86.79% (range 80.64%–89.87%) corresponding to a quality grade B while the long-term practice cohort (N=84) reached a weighted average (IQR) of 87.32% (quality grade B; range 85.62%-89.87%).

Volume censoring

-

Statistical modeling & inference

Model type and settings

Mass-univariate analysis of cortical surface parameters using linear regression models.

Effect(s) tested

Positive associations between higher levels of cortical folding and motor behavior as predicted by the literature.

Specify type of analysis: ☐ Whole brain ☐ ROI-based ☒ Both

Anatomical location(s)

Anatomical locations were defined based on peak MNI coordinates for preSMA/SMA obtained from our previous balance-training study (Taubert et al., 2010).

Statistic type for inference
(See Eklund et al. 2016)

Statistical inference of positive relationships between behavioural parameters and cortical curvature was performed across the whole cortex (exploratory analysis) with non-parametric permutation test (vertex-level T-statistics) and 5000 permutations.

Correction

p-values were considered significant at an FWE corrected threshold of $p < 0.05$ (vertex-level multiple comparison correction).

Models & analysis

n/a	Involved in the study
☒	☐ Functional and/or effective connectivity
☒	☐ Graph analysis
☒	☐ Multivariate modeling or predictive analysis
